# Rupture of a Superior Thyroid Artery Pseudoaneurysm After Tracheostomy: A Case Report

**DOI:** 10.7759/cureus.104739

**Published:** 2026-03-05

**Authors:** Keisuke Sasaguchi, Susumu Matsushime, Nobuichiro Tamura

**Affiliations:** 1 Department of Emergency Medicine, Kurashiki Central Hospital, Kurashiki, JPN

**Keywords:** airway hemorrhage, delayed hemorrhage, massive hemorrhage, pseudoaneurysm, superior thyroid artery, tracheostomy

## Abstract

Minor bleeding after tracheostomy is common, whereas massive hemorrhage is rare and can be catastrophic. We report an 80-year-old man who underwent surgical tracheostomy for prolonged ventilatory support after multiple trauma. The thyroid isthmus was partially divided and reflected caudally to create a tracheal window at the second to third tracheal rings. Hemostasis of the divided edge was achieved with electrocautery (without suturing or vessel ligation), and no pulsatile bleeding or significant vessel was encountered intraoperatively. During recovery, delirium and agitation led to frequent neck motion and marked tracheostomy tube movement. On hospital day 44, he developed sudden pulsatile bleeding from the stoma; temporary control was achieved with orotracheal intubation and local compression, and contrast-enhanced CT revealed a pseudoaneurysm arising from the right superior thyroid artery adjacent to the stoma. Despite initial observation in the absence of active extravasation, rebleeding occurred on hospital day 49 with airway flooding and cardiac arrest. After resuscitation, surgical ligation of the superior thyroid artery eliminated the pseudoaneurysm, but the patient ultimately died from hypoxic encephalopathy. This case highlights the need for prompt vascular evaluation and early definitive management when massive post-tracheostomy bleeding suggests an arterial pseudoaneurysm.

## Introduction

Tracheostomy is commonly performed to secure the airway in patients requiring prolonged mechanical ventilation or those with upper airway obstruction. However, it is associated with a wide range of early and late complications, including bleeding, infection, and tracheal stenosis [1,2]. Bleeding is one of the more frequent complications; in most cases, it presents as minor oozing from superficial vessels around the stoma and can be controlled with local compression or other conservative measures [1-3]. In the UK National Confidential Enquiry into Patient Outcome and Death (NCEPOD) report, tracheostomy-related bleeding was reported as minor in 4.4% and major in 1.2% of patients [4].

In contrast, massive hemorrhage after tracheostomy is uncommon but can be rapidly fatal due to airway flooding and circulatory collapse [1-3]. The most feared cause of delayed massive bleeding is tracheo-innominate artery fistula (TIF), which has been reported in approximately 0.1-1% patients after surgical tracheostomy [5]. Potential sources of massive bleeding also include injury to the brachiocephalic (innominate) artery and thyroid arteries; however, thyroid artery injury (including pseudoaneurysm formation) appears to be exceedingly rare and is described predominantly in isolated case reports and small case series, precluding reliable incidence estimates [6-8]. In addition to surgical hemostasis, endovascular approaches, such as transcatheter arterial embolization (TAE) and stent placement, have been reported as effective treatments for life-threatening tracheostomy-related hemorrhage [2,3,6,7].

In general, peripheral arterial pseudoaneurysms arise from disruption of the arterial wall due to trauma or iatrogenic injury, followed by persistent blood flow into a contained hematoma cavity; the lesion may gradually enlarge and eventually rupture [9]. Tracheostomy-related rupture of a superior thyroid artery pseudoaneurysm is exceedingly rare, with only a few cases reported in the literature [6-8].

Herein, we report a case of fatal superior thyroid artery pseudoaneurysm rupture with rebleeding following surgical tracheostomy performed for prolonged mechanical ventilation after multiple traumas. Considering the relevant literature, we describe the clinical course and discuss evaluation and management strategies for massive bleeding after tracheostomy.

## Case presentation

Patient information

**Figure 1:**
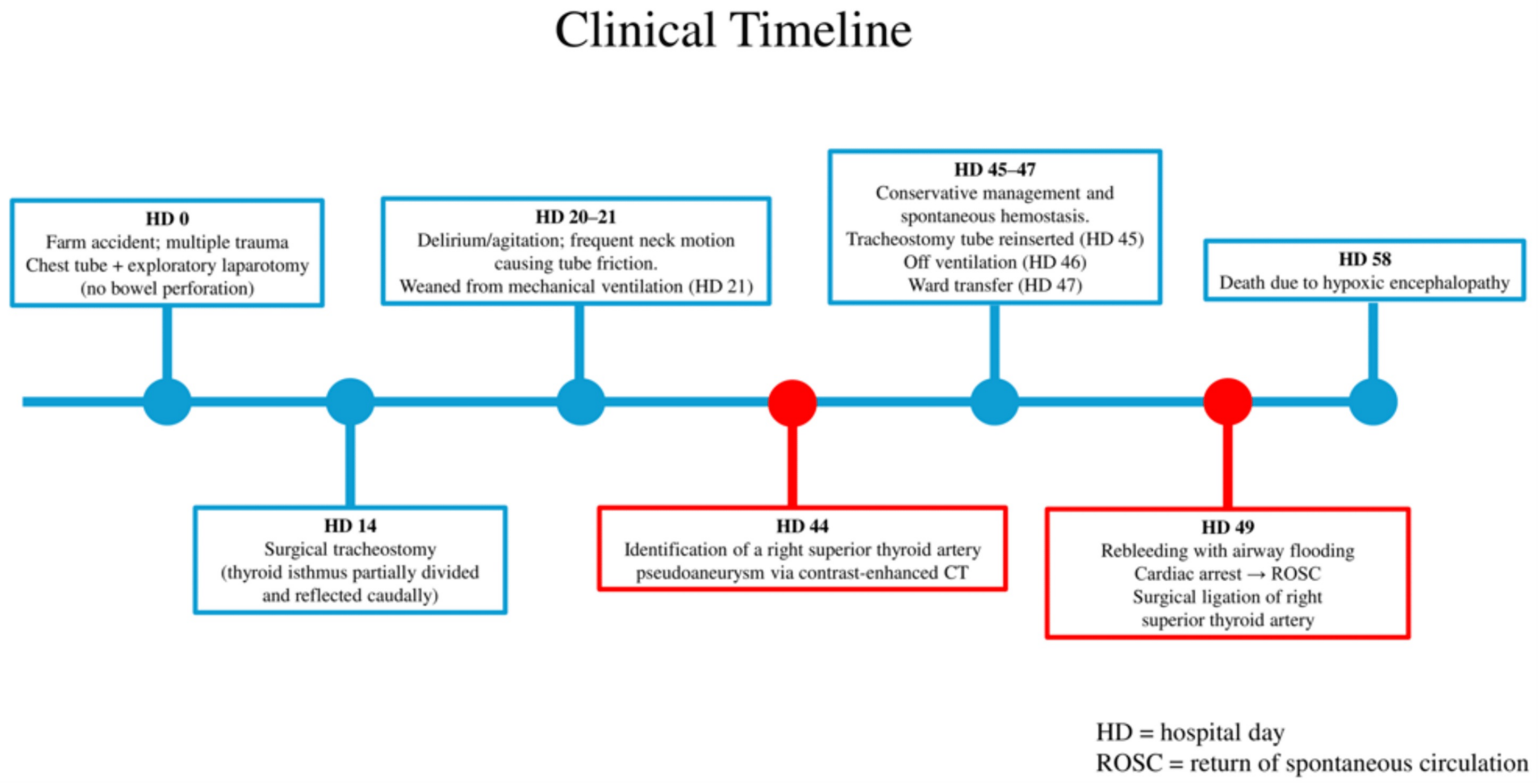
Clinical timeline of the case Blue markers indicate the baseline clinical course; red markers indicate major bleeding events.

An 80-year-old man with a medical history of hypertension and dyslipidemia, but no history of antiplatelet or anticoagulant therapy, was brought to our hospital after a farm accident. The patient’s clinical course is summarized in Figure 1.

Mechanism of injury and initial management

The patient fell from a tractor and was subsequently found with his trunk crushed beneath it. Upon arrival, his vital signs were as follows: blood pressure 140/80 mmHg; heart rate 96 beats/min; respiratory rate 24 breaths/min; and peripheral oxygen saturation (SpO₂) 95% on 6 L/min of oxygen via a face mask. Physical examination revealed paradoxical breathing, left chest pain, left flank pain, and burns on the right lower leg. Based on physical findings and imaging studies, he was diagnosed with multiple traumas, including flail chest due to multiple rib fractures, hemopneumothorax, lower extremity burns caused by entrapment under a tractor engine, and suspected small bowel perforation.

The patient underwent exploratory laparotomy on the day of admission for suspected small bowel perforation. Intraoperatively, a small amount of hemoperitoneum was observed; however, no obvious bowel perforation was noted. After surgery, the patient was admitted to the intensive care unit (ICU) for ongoing mechanical ventilation.

Tracheostomy

Because liberation from mechanical ventilation was delayed owing to flail chest and respiratory muscle fatigue, prolonged ventilatory support was anticipated. On hospital day 14, a surgical tracheostomy was performed. A midline cervical skin incision was made, and the strap muscles were separated in the midline to expose the thyroid isthmus. The isthmus was mildly enlarged, partially divided, and reflected caudally to ensure adequate exposure. Hemostasis of the divided edge was achieved with electrocautery without suturing of the isthmus, and no vessel ligation was performed. A tracheal window was then created at the level of the second and third tracheal rings, and a cuffed tracheostomy tube was inserted. No excessive bleeding occurred during the procedure, and no pulsatile bleeding or significant vessel was encountered in the operative field. Meticulous hemostasis was confirmed before completion.

Postoperative course and first episode of massive bleeding

Following the tracheostomy, the patient’s respiratory status remained stable, and sedation was gradually tapered. The patient was transferred out of the ICU on hospital day 16. Around hospital day 20, the patient developed delirium with agitation, posing a high risk for self-decannulation and line removal. The patient was managed with a combination of physical restraints and pharmacological treatment. Despite these challenges, the patient was successfully weaned off mechanical ventilation on hospital day 21, and a tracheostomy tube was left in place for airway management. Episodes of excessive neck flexion and extension, as well as marked movement of the tracheostomy tube, were frequently observed. Occasional small amounts of blood-tinged secretions were noted around the stoma; however, these were easily controlled with local compression and dressing changes, and the decrease in hemoglobin was minimal.

On hospital day 44, during a rehabilitation session, the patient suddenly developed massive, pulsatile bleeding from the tracheostomy site. Rehabilitation was immediately discontinued, and the patient was transferred back to bed. Orotracheal intubation was performed to secure the airway, and mechanical ventilation was resumed. The tracheostomy tube was removed, and finger pressure and gauze packing were applied to the tracheostomy site to achieve temporary hemostasis. Transient hypotension was observed; however, the patient’s hemodynamics promptly stabilized after fluid resuscitation and red blood cell transfusion.

Imaging findings and initial management strategy

**Figure 2:**
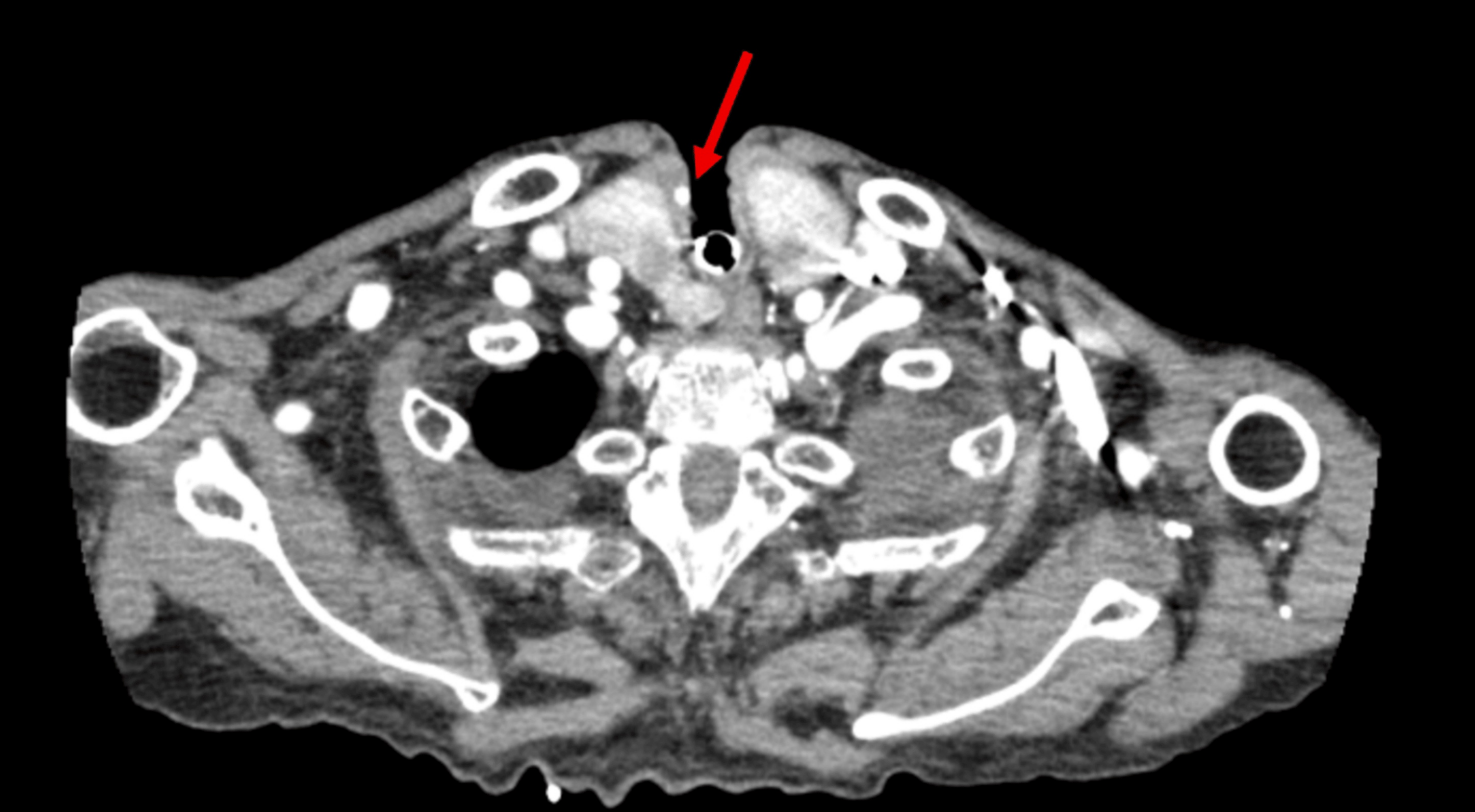
Contrast-enhanced CT showing a superior thyroid artery pseudoaneurysm after the first bleeding episode Axial arterial-phase contrast-enhanced CT performed after the first massive bleeding episode shows a focal enhancing lesion adjacent to the tracheostomy stoma, compatible with a pseudoaneurysm of the right superior thyroid artery (arrow).

After hemostasis was achieved, arterial-phase contrast-enhanced CT of the neck and upper mediastinum was performed. It revealed a small, intensely enhancing nodular lesion measuring 4.5 mm in maximal diameter in the territory of the right superior thyroid artery, located adjacent to the tracheostomy stoma (Figure 2). The lesion was situated near the area where the thyroid isthmus had been divided during tracheostomy and was considered a pseudoaneurysm arising from a branch of the superior thyroid artery.

No obvious contrast extravasation was observed on contrast-enhanced CT, and temporary hemostasis was clinically achieved with gauze compression. In addition, the suspected pseudoaneurysm was extremely small (4.5 mm in maximal diameter), which was considered one of the reasons supporting our expectation of spontaneous thrombosis. Therefore, we chose close observation with continued local compression/packing and frequent reassessment, anticipating spontaneous thrombosis. Endovascular intervention (e.g., embolization) was not performed at that time. On hospital day 45, compression was released, hemostasis at the tracheostomy site was confirmed, and the tracheostomy tube was reinserted. Mechanical ventilation was discontinued on hospital day 46, and the patient was transferred to the general ward on hospital day 47.

Rebleeding, surgical hemostasis, and outcome

However, on hospital day 49, he developed massive bleeding from both the tracheostomy site and the oral cavity. A large volume of blood rapidly entered the airways, leading to severe hypoxemia. Although reintubation was attempted, the patient progressed to cardiac arrest. Spontaneous circulation was achieved after cardiopulmonary resuscitation, and the patient was readmitted to the ICU.

**Figure 3:**
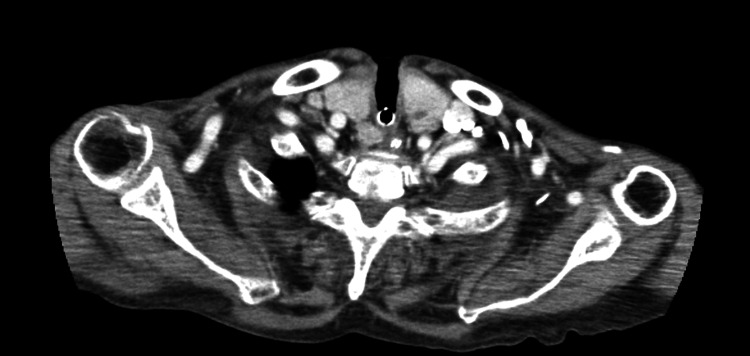
Postoperative contrast-enhanced CT demonstrating disappearance of the pseudoaneurysm after surgical ligation. Axial arterial-phase contrast-enhanced CT obtained after surgical ligation of the right superior thyroid artery shows complete resolution of the previously identified pseudoaneurysm adjacent to the tracheostomy stoma.

In the ICU, an otolaryngologist performed surgical ligation of the proximal right superior thyroid artery. Postoperative CT revealed the complete disappearance of the pseudoaneurysm (Figure 3). No further bleeding occurred thereafter. However, the patient developed hypoxic encephalopathy secondary to the cardiac arrest, and his neurological status did not improve. The patient’s overall condition gradually deteriorated, and he died on hospital day 58 from hypoxic encephalopathy.

## Discussion

This case report describes a rare but devastating complication of tracheostomy: rupture and re-rupture of a superior thyroid artery pseudoaneurysm occurring approximately one month after surgical tracheostomy in a patient requiring prolonged mechanical ventilation following multiple traumas. Reports of tracheostomy-related pseudoaneurysms involving the superior thyroid artery (or thyroid artery) are limited. To the best of our knowledge, previously reported cases include a pseudoaneurysm following revision tracheostomy reported by Lin and Reilly [8]; a pseudoaneurysm after surgical tracheostomy in a burn patient reported by Tanaka et al. [6]; and three cases of tracheostomy-site bleeding managed with thyroid artery embolization reported by Park et al. [7]. In all these cases, the culprit vessel was identified by contrast-enhanced CT or angiography, and transcatheter arterial embolization (TAE) was successfully performed, resulting in favorable outcomes [6-8]. In contrast, our patient experienced fatal rebleeding after initial pseudoaneurysm rupture, representing, to our knowledge, an extremely rare outcome among tracheostomy-related superior thyroid artery pseudoaneurysm cases.

Mechanism of pseudoaneurysm formation

Pseudoaneurysms of peripheral arteries, including the superior thyroid artery, are relatively uncommon but have been increasingly reported to be associated with iatrogenic arterial injury due to catheterization, surgery, and thermal ablation procedures [9-11]. Pseudoaneurysms arising from the thyroid arteries have been described in the thyroid and parathyroid regions following fine-needle aspiration, radiofrequency ablation, and inadvertent vascular puncture [10,11]. Because the neck contains vital vascular and aerodigestive structures in close proximity, hemorrhage in this region can rapidly become life-threatening.

In general, pseudoaneurysms form when full-thickness disruption of the arterial wall occurs due to trauma or iatrogenic injury. Blood continues to flow into the surrounding hematoma cavity containing perivascular tissues, with persistent arterial inflow promoting expansion and eventual rupture [9]. In our case, several factors likely contributed to the pseudoaneurysm formation and enlargement, including partial division of the thyroid isthmus during tracheostomy; formation of a pseudoaneurysm in the superior thyroid artery territory adjacent to the tracheostomy stoma suggesting minor intraoperative injury to the superior thyroid artery or one of its branches; agitation and delirium leading to difficulty maintaining bed rest with frequent excessive neck flexion and extension and substantial movement of the tracheostomy tube; and possible tissue fragility around the long-term indwelling tracheostomy tube due to chronic inflammation or infection.

These factors may have acted synergistically to promote pseudoaneurysm formation and subsequent rupture.

Management considerations: limitations of conservative treatment

In this case, although a superior thyroid artery pseudoaneurysm adjacent to the tracheostomy stoma was suspected after the first episode of massive bleeding, conservative management was chosen because contrast-enhanced CT showed no obvious contrast extravasation, and clinical hemostasis was achieved with compression. The lesion also appeared very small (4.5 mm in maximal diameter) and superficially located on CT, raising the expectation of spontaneous thrombosis. Conservative observation has been reported for selected small iatrogenic pseudoaneurysms in compressible locations (most commonly post-catheterization femoral pseudoaneurysms), with criteria favoring early intervention, including larger size (e.g., >3 cm), interval expansion, expanding hematoma, pain, infection, neurovascular compromise, or inability to comply with follow-up [12].

However, peripheral arterial pseudoaneurysms can enlarge and rupture through repeated cycles of thrombosis and recanalization [9]. Moreover, the applicability of observation criteria derived from compressible sites to pseudoaneurysms adjacent to the airway and tracheostomy stoma is uncertain. In this high-risk setting, even transient rebleeding can immediately cause airway flooding and circulatory collapse. Therefore, once a massive hemorrhage occurs and a pseudoaneurysm is identified, the risk of rebleeding should be considered as high regardless of temporary hemostasis or the absence of active extravasation on imaging. In our patient, re-rupture occurred five days after the initial bleeding episode, resulting in massive hemorrhage, airway flooding, and cardiac arrest.

Previous reports have recommended early vascular evaluation with contrast-enhanced CT and/or angiography for massive bleeding after tracheostomy, followed by definitive intervention (TAE or surgical ligation/reconstruction) once the responsible vessel has been identified [2,3,6-8]. Tracheostomy-related thyroid artery pseudoaneurysm is exceedingly rare, and most published cases have been treated definitively, typically with embolization, with favorable outcomes [6-8]. The thyroid arteries are relatively peripheral branches and are technically amenable to embolization [6,7,10,11]. In retrospect, unfamiliarity with this rare entity, together with the apparent small size and lack of extravasation on CT, contributed to an overly conservative strategy in our case. Had TAE or surgical hemostasis been performed at the time of the first massive bleeding episode, fatal rebleeding might have been prevented.

Practical considerations in patients with poor cooperation

Maintaining bed rest and stable positioning is often difficult in patients with delirium or cognitive impairment. Such patients are prone to displacement of the tracheostomy tube and excessive neck movement, which increases mechanical stress on the surrounding tissues. In our case, this situation may have exacerbated a minor intraoperative vascular injury and contributed to pseudoaneurysm formation and rupture.

Therefore, when performing a tracheostomy in patients who are expected to have difficulty cooperating or maintaining rest, it is important to choose a surgical approach that minimizes manipulation of the thyroid isthmus and adjacent vessels whenever possible; limit thermal injury through careful use of electrocautery; reinforce tracheostomy tube fixation; optimize postoperative neck positioning; and implement delirium prevention and management strategies, including environmental modification, appropriate pharmacological therapy, and early mobilization, to reduce excessive body and neck movements.

These multifaceted measures may help to reduce the risk of vascular injury, pseudoaneurysm formation, and delayed arterial hemorrhage around the tracheostomy site.

Clinical implications

From this case, we highlight two key clinical messages. First, in patients with a tracheostomy who experience even a single episode of massive bleeding, clinicians should suspect arterial hemorrhage and pseudoaneurysm formation, perform prompt vascular evaluation with contrast-enhanced CT and/or angiography, and prioritize definitive management with TAE or surgical hemostasis regardless of temporary hemostasis or the presence or absence of active contrast extravasation [2,3,6-8]. Second, in patients with delirium or agitation who have difficulty maintaining bed rest, meticulous attention should be paid to thyroid handling and tracheostomy tube fixation during and after tracheostomy, and efforts should be made to prevent excessive tube movement, as this may increase the risk of vascular injury and pseudoaneurysm formation.

## Conclusions

The rupture of a superior thyroid artery pseudoaneurysm after tracheostomy is an extremely rare and potentially fatal complication. In patients with poor cooperation, meticulous surgical techniques to minimize trauma to the thyroid gland and its vessels, as well as careful postoperative management of the tracheostomy tube, are essential. Furthermore, when a patient with a tracheostomy experiences even a single episode of massive bleeding, clinicians should suspect arterial injury, including a superior thyroid artery pseudoaneurysm, and promptly perform vascular imaging, followed by definitive intervention with TAE or surgical ligation, as appropriate. This case underscores the importance of early definitive treatment and highlights the fact that tracheostomy-related superior thyroid artery pseudoaneurysms can result in fatal outcomes.
